# Darcy–Forchheimer MHD Couple Stress 3D Nanofluid over an Exponentially Stretching Sheet through Cattaneo–Christov Convective Heat Flux with Zero Nanoparticles Mass Flux Conditions

**DOI:** 10.3390/e21090867

**Published:** 2019-09-06

**Authors:** Muhammad Wakeel Ahmad, Poom Kumam, Zahir Shah, Ali Ahmad Farooq, Rashid Nawaz, Abdullah Dawar, Saeed Islam, Phatiphat Thounthong

**Affiliations:** 1 Department of Mathematics, Abdul Wali Khan University, Mardan 23200, Pakistan; 2 KMUTT-Fixed Point Research Laboratory, Room SCL 802 Fixed Point Laboratory, Science Laboratory Building, Department of Mathematics, Faculty of Science, King Mongkut’s University of Technology Thonburi (KMUTT), 126 Pracha-Uthit Road, Bang Mod, Thrung Khru, Bangkok 10140, Thailand; 3 KMUTT-Fixed Point Theory and Applications Research Group, Theoretical and Computational Science Center (TaCS), Science Laboratory Building, Faculty of Science, King Mongkut’s University of Technology Thonburi (KMUTT), 126 Pracha-Uthit Road, Bang Mod, Thrung Khru, Bangkok 10140, Thailand; 4 Department of Medical Research, China Medical University Hospital, China Medical University, Taichung 40402, Taiwan; 5 Center of Excellence in Theoretical and Computational Science (TaCS-CoE), SCL 802 Fixed Point Laboratory, Science Laboratory Building, King Mongkut’s University of Technology Thonburi (KMUTT), 126 Pracha-Uthit Road, Bang Mod, Thrung Khru, Bangkok 10140, Thailand; 6 Mathematics Department, COMSATS University, Abbottabad Campus, Islamabad 22060, Pakistan; 7 Department of Mathematics, Qurtuba University of Science and Information Technology, Peshawar 25000, Pakistan; 8 Renewable Energy Research Centre, Department of Teacher Training in Electrical Engineering, Faculty of Technical Education, King Mongkut’s University of Technology North Bangkok, 1518 Pracharat 1 Road, Bangsue, Bangkok 10800, Thailand

**Keywords:** MHD, nanofluids, heat transfer, couple stress fluid, HAM, Cattaneo–Christov heat flux model

## Abstract

In the last decade, nanoparticles have provided numerous challenges in the field of science. The nanoparticles suspended in various base fluids can transform the flow of fluids and heat transfer characteristics. In this research work, the mathematical model is offered to present the 3D magnetohydrodynamics Darcy–Forchheimer couple stress nanofluid flow over an exponentially stretching sheet. Joule heating and viscous dissipation impacts are also discussed in this mathematical model. To examine the relaxation properties, the proposed model of Cattaneo–Christov is supposed. For the first time, the influence of temperature exponent is scrutinized via this research article. The designed system of partial differential equations (PDE’s) is transformed to set of ordinary differential equations (ODE’s) by using similarity transformations. The problem is solved analytically via homotopy analysis technique. Effects of dimensionless couple stress, magnetic field, ratio of rates, porosity, and coefficient of inertia parameters on the fluid flow in *x*- and *y*-directions have been examined in this work. The augmented ratio of rates parameter upsurges the velocity profile in the *x*-direction. The augmented magnetic field, porosity parameter, coefficient of inertia, and couple stress parameter diminishes the velocity field along the *x*-direction. The augmented magnetic field, porosity parameter, coefficient of inertia, ratio of rates parameter, and couple stress parameter reduces the velocity field along the *y*-axis. The influences of time relaxation, Prandtl number, and temperature exponent on temperature profile are also discussed. Additionally, the influences of thermophoresis parameter, Schmidt number, Brownian motion parameter, and temperature exponent on fluid concentration are explained in this work. For engineering interests, the impacts of parameters on skin friction and Nusselt number are accessible through tables.

## 1. Introduction

Nanofluids are used inside hybrid-powered machines, fuel cells, microelectronics, pharmaceutical procedures, and nanotechnologies. Choi [1] immersed nanoparticles into a base fluid for the first time. Wang and Mujumdar [2] prepared nanofluids by adding metallic and non-metallic nano-particles into base fluids and explained the heat transfer characteristics of the nanofluids. The study of Wang and Mujumdar was later numerically deliberated by Eastman et al. [3,4]. Tiwari and Das [5] designed a model for single-phase nanofluids, but, in contrast, Buongiorno [6] constructed the second-phase mathematical model for nanofluids. Soon after, numerous researchers have been conducted in diverse regions of interest regarding nanofluids. Kasaeian et al. [7] worked on the performance of heat transmission in nanofluid flow. Ramzan et al. [8] explored the radiative magnetohydrodynamic (MHD) flow of nanofluid. Sheikholeslami et al. [9] solved numerically the MHD nanofluid flow through a porous medium. Besthapu et al. [10] probed nanofluid mixed convection flow with MHD by observing the viscous dissipation impacts. Dawar et al. [11] scrutinized nanofluid flow over an unsteady oscillatory stretching sheet. Alharbi et al. [12] included the MHD effects and examined the entropy generation. Shah et al. [13] probed Darcy–Forchheimer nanofluid flow with inertial effect. Khan et al. [14] studded MHD flow of Darcy–Forchheimer nanofluid with the impact of thermal radiation. Zubair et al. [15] explored 3D Darcy–Forchheimer squeezing nanofluid flow with Cattaneo–Christov heat flux via entropy generation. Khan et al. [16] studded the flow of nanofluid past a linearly stretching surface. The MHD nanofluid flow via entropy generation with viscous dissipation impact was explored by Dawar et al. [17]. Sheikholeslami [18] explored free convective nanofluid in medium under effect of electric field. Sheikholeslami [19] investigated the flow of water-based nanofluid with Brownian motion magnetic field impacts. Dawar et al. [20] explored Darcy–Forchheimer flow of nanofluids over stretching surface analytically via convective conditions. Ramzan et al. [21] examined the heat transfer rate in couple stress MHD nanofluid flow.

In 1822, Fourier [22] designed a heat transmission model for the material. Later on, Cattaneo [23] modified the Fourier model by adding a term of thermal relaxation time. Afterwards Christov [24] further improved the Cattaneo model [23], called the Cattaneo–Christov heat flux model. Straughan [25] deliberated the stability of wave motion in a porous medium by applying the Cattaneo–Christov heat flux model. Straughan [26] investigated the characteristics of heat transmission in a nanofluid. Han et al. [27] explicated the thermal transmission in viscoelastic fluids. Khan et al. [28] numerically calculated [24] over an exponentially stretching surface. Hayat et al. [29] deliberated various features of advanced mass and the heat flux model of the nanofluid flow. Tibullo et al. [30] probed the model of [24] for incompressible fluids. Ciarletta et al. [31] constructed a stability and uniqueness model for [24]. Haddad [32] examined thermal stability for model [24] in porous medium. Mustafa [33] took model [24] and explained it for heat transfer in a rotating flow of nanofluid. Hayat et al. [34] investigated impacts of model [24] during the flow of various fluids. Waqas et al. [35] assumed Burger’s fluids thermal conductivity by taking model [24]. Zheng et al. [36] investigated the viscoelastic MHD fluid flow and heat transmission past a stretching sheet by applying model [24]. Shah et al. [37] explored MHD flow of an electrically-conducting ferrofluid by taking model [24] over a stretching surface. Hayat et al. [38] took model [24] and probed 3D nanofluid flow over a stretching surface. Muskat et al. [39] explained the nature of homogeneous fluid flow through a porous medium. Seddeek et al. [40] assumed the Darcy–Forchheimer model and studded the flow of mixed convention fluid with the effects of viscous dissipation and thermophoresis. Pal et al. [41] explored the nature of fluid flow in a porous medium by taking Darcy–Forchheimer model. Sadiq et al. [42] explored the MHD flow of the Maxwell nanofluid through a heated sheet by assuming the Darcy–Forchheimer model. Wakif et al. [43] numerically examined the nanofluid flow with external magnetic field. Wakif et al. [44] examined the MHD nanofluid flow with thermal radiation impact. Wakif et al. [45] scrutinized the water-based nanofluid with uniform magnetic field impact. The other related studies of Wakif et al. can be seen in [46,47,48,49]. Zubiar et al. [50] studied entropy generation in a squeezing nanofluid flow.

The main aim of this investigation is to study the 3D magnetohydrodynamic flow of Darcy–Forchheimer couple stress nanofluid over a porous exponentially stretching film. The present work is done with joule heating and viscous dissipation effects. To examine the thermal relaxation time, the Cattaneo–Christov model of heat flux is applied. For the first time, the influences of the temperature exponent are explored through this research article.

## 2. Problem Formulation

**Figure 1:**
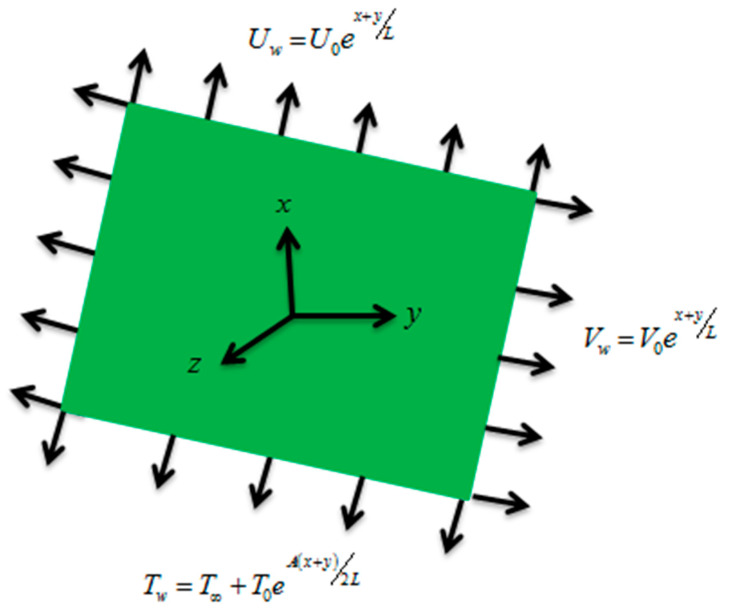
Geometrical representation of the flow.

Assume 3D couple stress nanofluids flow over a porous exponentially stretching sheet, having null mass flux and convection of heat conditions. Stretching velocity in the *x*-direction is of the form u=Uw(x,y)=U0ex+yL, while velocity in the *y*-direction is to be assumed as v=Vw(x,y)=V0ex+yL, here $\left(U_{0},V_{0}\right)$ are constants (Figure 1). The joule heating, viscous dissipation, and impacts of uniform magnetic field are applied in the present nanofluids flow model. A uniform magnetic field is assumed in the *z*-direction. The temperature of the porous stretching surface will be kept at continual temperature $T_{w}$, while surroundings temperature is $T_{\infty }$, constant concentration dented by $C_{w}$ and $C_{\infty }$ shows ambient concentration.

The governing equations of the modeled problem are [21,38]:

(1) $$\frac{\partial u}{\partial x}+\frac{\partial v}{\partial y}+\frac{\partial w}{\partial z}=0,$$

(2) $$u\frac{\partial u}{\partial x}+v\frac{\partial u}{\partial y}+w\frac{\partial u}{\partial z}=\nu \frac{\partial^{2}u}{\partial z^{2}}-\nu^{\prime }\frac{\partial^{4}u}{\partial z^{4}}-\frac{\sigma B_{0}^{2}}{\rho }u-\left(\frac{\nu }{k}+Fu\right)u,$$

(3) $$u\frac{\partial v}{\partial x}+v\frac{\partial v}{\partial y}+w\frac{\partial v}{\partial z}=\nu \frac{\partial^{2}v}{\partial z^{2}}-\nu^{\prime }\frac{\partial^{4}v}{\partial z^{4}}-\frac{\sigma B_{0}^{2}}{\rho }v-\left(\frac{\nu }{k}+Fv\right)v,$$

(4) $$\rho c_{p}\left(u\frac{\partial T}{\partial x}+v\frac{\partial T}{\partial y}+w\frac{\partial T}{\partial z}\right)=-\nabla \cdot \vec{q},$$

(5) $$u\frac{\partial C}{\partial x}+v\frac{\partial C}{\partial y}+w\frac{\partial C}{\partial z}=D_{B}\left(\frac{\partial^{2}C}{\partial z^{2}}\right)+\frac{D_{T}}{T_{\infty }}\left(\frac{\partial^{2}T}{\partial z^{2}}\right),$$

The heat flux $\vec{q}$ satisfies

(6) $$\vec{q}+\lambda_{e}\left(\frac{\partial \vec{q}}{\partial t}+\vec{V}\cdot \nabla \vec{q}-\vec{q}\cdot \nabla \vec{V}+\left(\nabla \cdot \vec{V}\right)\vec{q}\right)=-\lambda_{1}\nabla T,$$

where $\lambda_{e}\;\mathrm{and}\;\lambda_{1}$ signify the thermal relaxation time and thermal conductivity, respectively. By taking $\lambda_{e}=0$, Equation (6) reduced to Fourier’s law. Furthermore eliminating $\vec{q}$ from Equations (4) and (6), we obtained heat equation as under:

(7) $$\begin{array}{l} u\text{​}\frac{\partial Τ}{\partial x}\text{​}+\text{​}v\text{​}\frac{\partial Τ}{\partial y}+\text{​}w\text{​}\;\frac{\partial Τ}{\partial z}=\frac{\lambda_{1}}{\rho c_{p}}\left(\frac{\partial^{2}Τ}{\partial z^{2}}\right)\\ -\lambda_{e}[\begin{array}{l} u^{2}\frac{\partial^{2}Τ}{\partial x^{2}}+v^{2}\frac{\partial^{2}Τ}{\partial y^{2}}+w^{2}\frac{\partial^{2}Τ}{\partial z^{2}}+2uv\frac{\partial^{2}Τ}{\partial x\partial y}+2vw\frac{\partial^{2}Τ}{\partial y\partial z}+2uw\frac{\partial^{2}Τ}{\partial x\partial z}\\ +\left(u\frac{\partial u}{\partial x}+v\frac{\partial u}{\partial y}+w\frac{\partial u}{\partial z}\right)\frac{\partial Τ}{\partial x}+\left(u\frac{\partial v}{\partial x}+v\frac{\partial v}{\partial y}+w\frac{\partial v}{\partial z}\right)\frac{\partial Τ}{\partial y}+\left(u\frac{\partial w}{\partial x}+v\frac{\partial w}{\partial y}+w\frac{\partial w}{\partial z}\right)\frac{\partial Τ}{\partial z} \end{array}], \end{array}$$

with boundary conditions

(8) $$\begin{array}{l} u\;=\;U_{w}\left(x,y\right)=U_{0}e^{\frac{x+y}{L}},\;v=V_{w}\left(x,y\right)=V_{0}e^{\frac{x+y}{L}},\;w=0,\;k\frac{\partial T}{\partial z}=-h_{f}\left(T_{w}-T\right),\;\\ \;D_{B}\frac{\partial C}{\partial z}+\frac{D_{T}}{T_{\infty }}\frac{\partial T}{\partial x}=0,\;\mathrm{at}\;z=0,\\ u\to \;0,\;v\to \;0,\;C\to \;C_{\infty },\;T\to \;T_{\infty }\;\;\mathrm{as}\;z\to \;\infty . \end{array}$$

The exceeding equations have velocity components u, v, w along their respective directions, ν shows kinematic viscosity, k indicates thermal conductivity, $F=C_{b}/\sqrt{B}x$ is the inertial coefficient of permeable medium, $\nu^{\prime }=\frac{n}{p}$ is the couple stress viscosity, where n defines viscosity parameter, σ is the electric charge density, ρ is the density, $h_{f}$ is the heat transfer coefficient, A is the temperature exponent, $C_{p}$ indicates specific heat, $D_{B}$ is the Brownian diffusion coefficient, $D_{T}$ is the thermophoresis diffusion coefficient, and L is the reference length.

Applying the following similarity transformations techniques [21]

(9) $$\begin{array}{l} u=U_{0}\;e^{\frac{x+y}{L}}f^{\prime },\;\;v=U_{0}e^{\frac{x+y}{L}}g^{\prime },\;\;w=-{\left(\frac{\nu \;U_{0}}{2L}\right)}^{\frac{1}{2}}e^{\frac{x+y}{2L}}\left(f+\xi f^{\prime }+g+\xi g^{\prime }\right),\\ T_{w}=T_{\infty }+T_{0}e^{\frac{A\left(x+y\right)}{2L}}\theta ,\;\;C_{w}=C_{\infty }+C_{0}e^{\frac{A\left(x+y\right)}{2L}}\phi ,\;\xi ={\left(\frac{\;U_{0}}{2\nu L}\right)}^{\frac{1}{2}}e^{\frac{x+y}{2L}}z. \end{array}$$

Equation (1) is gratified inexorably, and Equations (2)–(7) yield

(10) $$f^{\prime \prime \prime }-2(f^{\prime }+g^{\prime })f^{\prime }+(f+g)\;f^{\prime \prime }-Kf^{v}-(M^{2}+\kappa +Frf^{\prime })f^{\prime }=0,$$

(11) $$g^{\prime \prime \prime }-2(f^{\prime }+g^{\prime })g^{\prime }+(f+g)g^{\prime \prime }-Kg^{v}-\left(M^{2}+\kappa +Fr\;g^{\prime }\right)g^{\prime }=0,$$

(12) $$\begin{array}{l} \frac{1}{\mathrm{Pr}}\theta^{\prime \prime }-A\left(f^{\prime }\;+g^{\prime }\right)\;\theta \;+\left(\;f+g\;\right)\theta^{\prime }+\\ \frac{\Lambda }{2}\left[\begin{array}{l} \left\{\xi \left(\text{​}f^{\prime }+g^{\prime }\text{​}\right)\text{​}+\text{​}\left(1+2A\right)\left(f+g\right)\right\}\left(f^{\prime }+g^{\prime }\right)\;\theta^{\prime }\\ -A\left\{\left(A+2\right){\left(f^{\prime }+g^{\prime }\right)}^{2}-\left(f+g\right)\left(f^{\prime \prime }\;+\;g^{\prime \prime }\right)\;\right\}\theta -{\left(f\;+g\;\right)}^{2}\theta^{\prime \prime } \end{array}\right]=0, \end{array}$$

(13) $$\phi^{\prime \prime }-ScA\left(f^{\prime }+g^{\prime }\right)\phi +Sc\left(f+g\right)\phi^{\prime }+\frac{Nt}{Nb}\theta^{\prime \prime }=0,$$

Satisfying the following boundary conditions

(14) $$\begin{array}{l} f\;=\;0,\;f^{\prime }\;=\;1,\;g\;=\;0,\;g^{\prime }\;=\;\alpha ,\;\theta^{\prime }=\;-\gamma \;\left(1-\theta \right),\;Nb\phi^{\prime }\;+\;Nt\theta^{\prime }=\;0\;\mathrm{at}\;\;\xi =\;0,\\ f^{\prime }\to \;0,\;g^{\prime }\to \;0,\;\theta \;\to \;0,\;\phi \;\to \;0\;\mathrm{as}\;\;\xi \;\to \;\infty . \end{array}$$

In Equations (9)–(13), $K=\frac{\nu^{\prime }a}{\nu^{2}}$ indicates dimensionless couple stress parameter, $M^{2}=\frac{2\sigma B_{0}^{2}L}{\rho U_{w}}$ represents the Hartmann number, $\alpha =\frac{V_{0}}{U_{0}}$ indicates the quotient of rates parameter, $\kappa =\frac{2\nu L}{kU_{w}}$ indicates the porosity parameter, $Fr=\frac{2\nu C_{b_{}}}{\sqrt{B}x}$ indicates the coefficient of inertia, $\mathrm{Pr}=\frac{\nu \rho c_{p}}{\lambda_{1}}$ represents the Prandtl number, $\Lambda =\frac{\lambda_{e}U_{w}}{L}$ represents dimensionless thermal relaxation time,$Sc=\frac{\nu }{D_{B}}$ represents Schmidt number, $\gamma =\frac{h}{k}\sqrt{\frac{2\nu L}{U_{w}}}$ indicates the Biot number, $Nb=\frac{\tau D_{B}}{\nu }\left(C_{w}-C_{\;\infty }\right)$ represents the Brownian motion parameter, and $Nt=\frac{\tau D_{T}\left(T_{w}-T_{\infty }\right)}{\nu T_{\infty }}$ represents thermophoresis parameter.

(15) Cfx(Rex2)=e3(x+y)2Lf″(0),Cfy(Rex2)=e3(x+y)2Lg″(0),

## 3. Solution by HAM

Homtopy Analysis method (HAM) is applied to solve Equations (10)–(13) with boundary condition (14).

The initial guesses are assumed as:

(16) $$f_{0}(\xi )=1-e^{-\xi }{,\;g}_{0}(\xi )=\alpha \left(1-e^{-\xi }\right),\theta_{0}(\xi )=\left(\frac{\gamma }{\gamma +1}\right)e^{-\xi },\phi_{0}(\xi )=-\left(\frac{Nt}{Nb}\frac{\gamma }{\gamma +1}\right)e^{-\xi }.$$

$L_{f},L_{g},L_{\theta }$, and $L_{\phi }$ are selected as:

(17) $$L_{f}(f)=f^{\prime \prime \prime }-f^{\prime },L_{g}(g)=g^{\prime \prime \prime }-g^{\prime },L_{\theta }(\theta )=\theta^{\prime \prime }-\theta ,L_{\phi }(\phi )=\phi^{\prime \prime }-\phi ,$$

with the following resultant characteristics:

(18) $$\begin{array}{l} L_{f}\left(k_{1}+k_{2}e^{-\xi }+k_{3}e^{\xi }\right)=0,{\;L}_{g}\left(k_{4}+k_{5}e^{-\xi }+k_{6}e^{\xi }\right)=0,\\ L_{\theta }\left(k_{7}e^{-\xi }+k_{8}e^{\xi }\right)=0,{\;L}_{\phi }\left(k_{9}e^{-\xi }+k_{10}e^{\xi }\right)=0, \end{array}$$

Here $k_{i}(i=1,2,3,\ldots ,10)$ represents real constants in general solution of the modeled problem.

The consequential non-linear operators $N_{f\;},N{\text{​}}_{g},N_{\theta }\;\mathrm{and}\;N_{\phi }$ are specified as under:

(19) $$\begin{array}{l} N_{f}\left[f(\xi ;ℜ),g(\xi ;sℜ)\right]=\frac{d^{3}f(\xi ;ℜ)}{d\xi^{3}}-2\left(\frac{df(\xi ;ℜ)}{d\xi }+\frac{dg(\xi ;ℜ)}{d\xi }\right)\frac{df(\xi ;ℜ)}{d\xi }\\ +\left(f(\xi ;ℜ)+g(\xi ;ℜ)\right)\frac{d^{2}f(\xi ;ℜ)}{d\xi^{2}}-K\frac{d^{5}f(\xi ;ℜ)}{d\xi^{5}}-\left\{M^{2}+\kappa +Fr\frac{df(\xi ;ℜ)}{d\xi }\right\}\frac{df(\xi ;ℜ)}{d\xi }, \end{array}$$

(20) $$\begin{array}{l} N_{g}\left[g(\xi ;ℜ),f(\xi ;ℜ)\right]=\frac{d^{3}g(\xi ;ℜ)}{d\xi^{3}}-2\left(\frac{df(\xi ;ℜ)}{d\xi }+\frac{dg(\xi ;ℜ)}{d\xi }\right)\frac{dg(\xi ;ℜ)}{d\xi }\\ +\left(f(\xi ;ℜ)+g(\xi ;ℜ)\right)\frac{d^{2}g(\xi ;ℜ)}{d\xi^{2}}-K\frac{d^{5}g(\xi ;ℜ)}{d\xi^{5}}-\left\{M^{2}+\kappa +Fr\frac{dg(\xi ;ℜ)}{d\xi }\right\}\frac{dg(\xi ;ℜ)}{d\xi }, \end{array}$$

(21) $$\begin{array}{l} N_{\theta }\left[\theta (\xi ;ℜ),f(\xi ;\tau ),g(\xi ;ℜ)\right]=\frac{1}{\mathrm{Pr}}\frac{d^{2}\theta (\xi ;ℜ)}{d\xi^{2}}-A\left(\frac{df(\xi ;ℜ)}{d\xi }+\frac{dg(\xi ;ℜ)}{d\xi }\right)\theta (\xi ;ℜ)\\ +\left(f(\xi ;ℜ)+g(\xi ;ℜ)\right)\text{​}\frac{d\theta (\xi ;ℜ)}{d\theta }+\frac{\Lambda }{2}[\left\{\begin{array}{l} \xi \left(\frac{df(\xi ;ℜ)}{d\xi }+\frac{dg(\xi ;ℜ)}{d\xi }\right)\\ +\left(1+2A\right)\left(\begin{array}{l} f(\xi ;ℜ)\\ +g(\xi ;ℜ) \end{array}\right) \end{array}\right\}\left(\begin{array}{l} \frac{df(\xi ;ℜ)}{d\xi }\\ +\frac{dg(\xi ;ℜ)}{d\xi } \end{array}\right)\frac{d\theta (\xi ;ℜ)}{d\xi }\\ -A\left\{\left(A+2\right){\left(\frac{df(\xi ;ℜ)}{d\xi }+\frac{dg(\xi ;ℜ)}{d\xi }\right)}^{2}-\left(\begin{array}{l} f(\xi ;ℜ)+\\ g(\xi ;ℜ) \end{array}\right)\left(\frac{d^{2}f(\xi ;ℜ)}{d\xi^{2}}+\frac{d^{2}g(\xi ;ℜ)}{d\xi^{2}}\right)\right\}\theta (\xi ;ℜ)\\ -{\left(f(\xi ;ℜ)+g(\xi ;ℜ)\right)}^{2}\frac{d^{2}\theta (\xi ;ℜ)}{d\xi^{2}}], \end{array}$$

(22) $$\begin{array}{l} N_{\Phi }\left[\Phi (\xi ;ℜ),f(\xi ;ℜ),\;g(\xi ;ℜ),\;\theta (\xi ;\;ℜ\;)\right]=\;\frac{d^{2}\Phi (\xi ;ℜ)}{d\xi^{2}}-\\ ScA\left(\frac{df(\xi ;ℜ)}{d\xi }+\frac{dg(\xi ;ℜ)}{d\xi }\right)\Phi (\xi ;ℜ)+Sc\;\cdot (f(\xi ;ℜ)+g(\xi ;ℜ))\;\frac{d\Phi (\xi ;ℜ)}{d\xi }\;+\;\frac{Nt}{Nb}\frac{d^{2}\theta (\xi ;ℜ)}{d\xi^{2}}, \end{array}$$

The zeroth-order problems from Equations (9)–(15) are:

(23) $$(1-ℜ)L_{f}\left[f(\xi ;ℜ)-f_{0}(\xi )\right]=ℜ\hbar_{f}N_{f}\left[f(\xi ;ℜ),g(\xi ;ℜ)\right],$$

(24) $$(1-ℜ)L_{g}\left[g(\xi ;ℜ)-g_{0}(\xi )\right]=ℜ\hbar_{g}N_{g}\left[g(\xi ;ℜ),f(\xi ;ℜ)\right],$$

(25) $$(1-ℜ)L_{\theta }\left[\theta (\xi ;ℜ)-\theta_{0}(\xi )\right]=ℜ\hbar_{\theta }N_{\theta }\left[\theta (\xi ;ℜ),f(\xi ;ℜ),g(\xi ;ℜ)\right],$$

(26) $$(1-ℜ)L_{\Phi }\left[\Phi_{p}(\xi ;ℜ)-\Phi_{0}(\xi )\right]=ℜ\hbar_{\Phi }N_{\Phi }\left[\Phi (\xi ;ℜ),f(\xi ;ℜ),g(\xi ;ℜ),\theta (\xi ;ℜ)\right],$$

The equivalent boundary conditions are:

(27) $$\begin{array}{l} {f(\xi ;ℜ)|}_{\xi =0}=0{\;\;\;\;\;\;\;\;\;\;\frac{df(\xi ;ℜ)}{d\xi }|}_{\xi =0}=1\;\;\;\;\;\;\;\;\;\;\;\;\;{\frac{df(\xi ;ℜ)}{d\xi }|}_{\xi \to \infty }=0\\ {\;{g(\xi ;\text{​}ℜ)|}_{\xi \text{​}=0}=0\;\;\;\;\;\;\;\;\;\;\;\frac{dg(\xi ;ℜ)}{d\xi }|}_{\xi =0}=\alpha \;\;{\;\;\;\;\;\;\;\;\;\;\frac{dg(\xi ;ℜ)}{d\xi }|}_{\xi \to \infty }\;=0\\ {\theta (\xi ;ℜ)|}_{\xi \to \infty }=0\;\;\;\;\;\;\;\;\;\;\;\;\;{\frac{d\theta (\xi ;\text{​}ℜ)}{d\xi }|}_{\xi =0}=-\gamma \left(1-\theta (\xi ;ℜ)\right)\;\;\;\;\;\;\;\;\;\;\;\;\\ {\;{\Phi (\xi ;ℜ)|}_{\xi \to \infty }=0\;\;\;\;\;\;\;\;\;Nb\frac{d\Phi (\xi ;\text{​}ℜ)}{d\xi }|}_{\xi =0}=-{Nt\;\frac{d\theta (\xi ;\text{​}ℜ)}{d\text{​}\xi }|}_{\xi =0} \end{array}$$

When ℜ=0 and ℜ=1 we have:

(28) $$\begin{array}{l} f(\xi ;0)=f_{0}(\xi ),\;\;\;f(\xi ;1)=f(\xi ),\\ g(\xi ;0)=g_{0}(\xi ),\;\;\;g(\xi ;1)=g(\xi ),\\ \theta (\xi ;0)=\theta_{0}(\xi ),\;\;\;\theta (\xi ;1)=\theta (\xi )\;,\\ \Phi (\xi ;0)=\Phi_{0}(\xi ),\;\;\;\Phi (\xi ;1)=\Phi (\xi )\;. \end{array}$$

By Taylor’s series expansion f(ξ;ℜ),g(ξ;ℜ),θ(ξ;ℜ) and ϕ(ξ;ℜ) can be written as:

(29) $$\begin{array}{l} f(\xi ;ℜ)=f_{0}(\xi )+\sum_{q=1}^{\infty }f_{q}(\xi ){ℜ}^{q},\;g(\xi ;ℜ)=\;g_{0}(\xi )+\sum_{q=1}^{\infty }g_{q}(\xi ){ℜ}^{q},\\ \theta (\xi ;ℜ)=\theta_{0}(\xi )+\sum_{q=1}^{\infty }\theta_{q}(\xi )\;{ℜ}^{q},\;\Phi (\xi ;ℜ)=\Phi_{0}(\xi )+\sum_{q=1}^{\infty }\Phi_{q}(\xi ){ℜ}^{q}. \end{array}$$

where

(30) $$\begin{array}{l} f_{q}(\xi )={\frac{1}{q!}\frac{df(\xi ;ℜ)}{d\xi }|}_{ℜ=0},\;g_{q}(\xi )={\frac{1}{q!}\frac{dg(\xi ;ℜ)}{d\xi }|}_{ℜ=0},\\ \theta_{q}(\xi )={\frac{1}{q!}\frac{d\theta (\xi ;ℜ)}{d\xi }|}_{ℜ=0}\;\mathrm{and}\;\Phi_{q}(\xi )={\frac{1}{q!}\frac{d\Phi (\xi ;ℜ)}{d\xi }|}_{ℜ=0}. \end{array}$$

The secondary constraints $\hbar_{f},\hbar_{g},\hbar_{\theta }\mathrm{and}\hbar_{\Phi }$ are chosen in such a way that the series in (29) becomes a convergent series at ℜ=1, by changing ℜ=1 in (29), we get:

(31) $$\begin{array}{l} f(\xi )=f_{0}(\xi )+\sum_{q=1}^{\infty }f_{q}(\xi ),\;g(\xi )=g_{0}(\xi )+\sum_{q=1}^{\infty }g_{q}(\xi ),\\ \theta (\xi )=\theta_{0}(\xi )+\sum_{q=1}^{\infty }\theta_{q}(\xi ),\;\Phi (\xi )=\Phi_{0}(\xi )+\sum_{q=1}^{\infty }\Phi_{q}(\xi ). \end{array}$$

For $q^{th}-$ order solution of the problem:

(32) $$\begin{array}{l} L_{f}\left[f_{q}(\xi )-\chi_{q}f_{q-1}(\xi )\right]=\hbar_{f}U_{q}^{f}(\xi ),\;g_{g}\left[g_{q}(\xi )-\chi_{q}g_{q-1}(\xi )\right]=\hbar_{g}U_{q}^{g}(\xi ),\\ L_{\theta }\left[\theta_{q}(\xi )-\chi_{q}\theta_{q-1}(\xi )\right]=\hbar_{\theta }U_{q}^{\theta }(\xi ),\;L_{\Phi }\left[\Phi_{\Phi }(\xi )-\chi_{q}\Phi_{q-1}(\xi )\right]=\hbar_{\Phi }U_{q}^{\Phi }(\xi ). \end{array}$$

The equivalent boundary conditions are:

(33) $$\begin{array}{l} f_{q}(0)\;=\;{f^{\prime }}_{q}(0)\;=\;{f^{\prime }}_{q}(\infty )\;=0,\\ g_{q}(0)\;=\;{g^{\prime }}_{q}(0)\;=\;{g^{\prime }}_{q}(\infty )\;=\;0,\\ {\theta^{\prime }}_{q}(0\;)\;=\;\theta_{q}\;(\infty )\;=\;0\\ \Phi_{q}(\;0\;)\;=\;\Phi_{q}(\infty )=0, \end{array}$$

Here

(34) $$U_{q}^{f}(\xi )={f^{\prime \prime \prime }}_{q-1}-2\sum_{k\;=0}^{q-1}\;\left({f^{\prime }}_{q-1}+{g^{\prime }}_{q-1}\right)f_{k}{}^{\prime }+\sum_{k=0}^{q-1}\left(f_{q-1}+g_{\;q-1}\right)f_{k}{}^{\prime \prime }-K\sum_{k=0}^{q-1}f_{q-1}^{v}-\sum_{k\;=0}^{q-1}\left\{\left(M^{2}+\kappa +Fr{f^{\prime }}_{q-1}\right){f^{\prime }}_{k}\right\},$$

(35) $$U_{q}^{g}(\xi )={g^{\prime \prime \prime }}_{q-1}-2\sum_{k\;=\;0}^{q-1}\left({f^{\prime }}_{q-1}+{g^{\prime }}_{q\;-\;1}\;\right){g^{\prime }}_{k}+\sum_{k\;=\;0}^{q-1}\left(f_{q-1}+\;\;g_{q\;-1}\right){g^{\prime \prime }}_{k}-K\sum_{k\;=0}^{q\;-1}g_{q-1}^{v}-\sum_{k=0}^{q-1}\left\{\;\left(M^{2}+\kappa +Fr\;{g^{\prime }}_{q-\;1}\right){g^{\prime }}_{k}\right\},$$

(36) $$\begin{array}{l} U_{q}^{\theta }(\xi )=\frac{1}{\mathrm{Pr}}{\theta^{\prime \prime }}_{q-1}-A\sum_{k=0}^{q-1}\left({f^{\prime }}_{q-1}+{g^{\prime }}_{q-1}\right)\theta_{k}+\sum_{k=\;0}^{q-1}\left(f_{q-1}+g_{q-1}\right){\theta^{\prime }}_{k}\\ +\frac{\Lambda }{2}[\left\{\xi \sum_{k=0}^{q-\text{​}1}\left({f^{\prime }}_{q-\text{​}1}+{g^{\prime }}_{q-\text{​}1}\right)+\;\left(1+2A\right)\;\sum_{k=0}^{q-1}\left(f_{q-\text{​}1}\;+\;g_{q-\text{​}1}\right)\right\}\;\sum_{k=0}^{q-\text{​}1}\left(\;{f^{\prime }}_{q-1}+{g^{\prime }}_{q-1}\right){\theta^{\prime }}_{k}\\ -A\left\{\left(A+2\right){\sum_{k=0}^{q-1}\left({f^{\prime }}_{q-\text{​}1}+{g^{\prime }}_{q-\text{​}1}\right)}^{2}+\sum_{k=0}^{q-1}\left(f_{q-1}+g_{q-1}\right)\left({f^{\prime \prime }}_{q-1}+{g^{\prime \prime }}_{q-1}\right)\right\}\theta_{k}-{\sum_{k=0}^{q-1}\left(f_{q-1}+g_{q-1}\right)}^{2}{\theta^{\prime \prime }}_{k}]\text{​} \end{array}$$

(37) $$U_{q}^{\Phi }(\xi )={\Phi^{\prime \prime }}_{q-1}-ScA\sum_{k=0}^{q-1}\left({f^{\prime }}_{q-1}+{g^{\prime }}_{q-1}\right)\Phi_{k}+Sc\sum_{k=0}^{q-1}\left(f_{q-1}+g_{q-1}\right){\Phi^{\prime }}_{k}+\frac{Nt}{Nb}{\theta^{\prime \prime }}_{q-1},$$

where

(38) $$\chi_{q}=\left\{ \begin{array}{l} 0,\;\mathrm{if}\;ℜ\leq 1\\ 1,\;\mathrm{if}\;ℜ>1 \end{array}\right..$$

## 4. HAM Convergence

**Figure 2:**
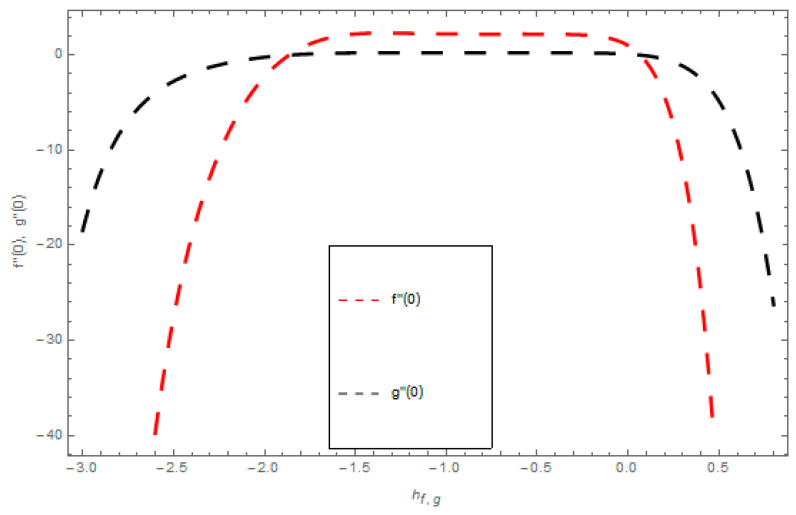
The ℏ-curves graph for velocity fields.

**Figure 3:**
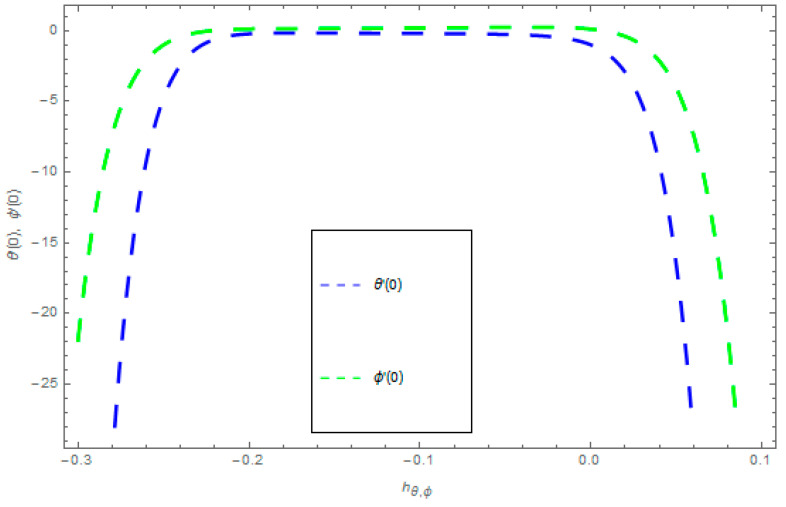
The ℏ-curves graph for temperature and concentration fields.

The velocity profiles convergence, temperature profile convergence, and concentration profile convergence are obtained through supporting parameters $\hbar_{f},\hbar_{g},\hbar_{\theta }$, and $\hbar_{\phi }$ of HAM are presented in Figure 2 and Figure 3. These legal ℏ-curves show the convergence regions for HAM.

## 5. Results and Discussion

**Figure 4:**
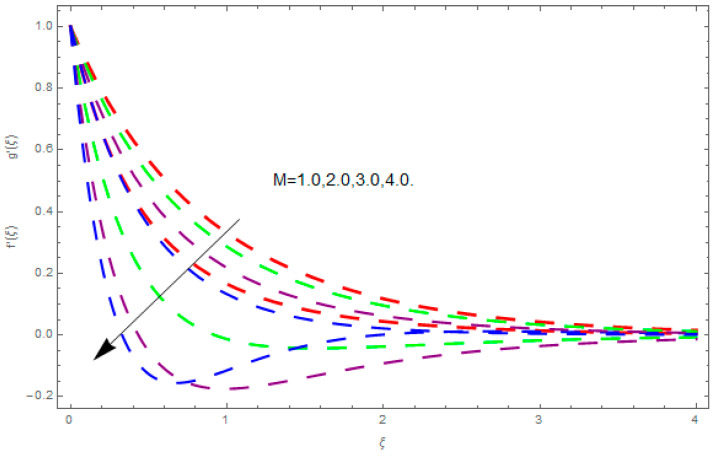
Impression of M on $f^{\prime }\left(\xi \right)$ and $g^{\prime }\left(\xi \right)$.

**Figure 5:**
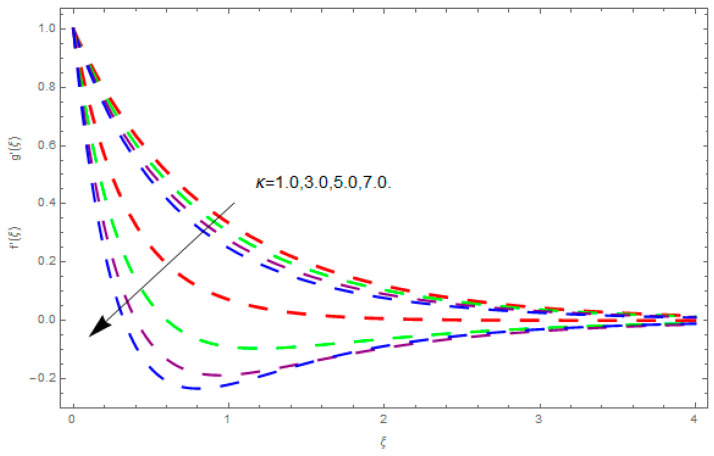
Impression of κ on $f^{\prime }\left(\xi \right)$ and $g^{\prime }\left(\xi \right)$.

**Figure 6:**
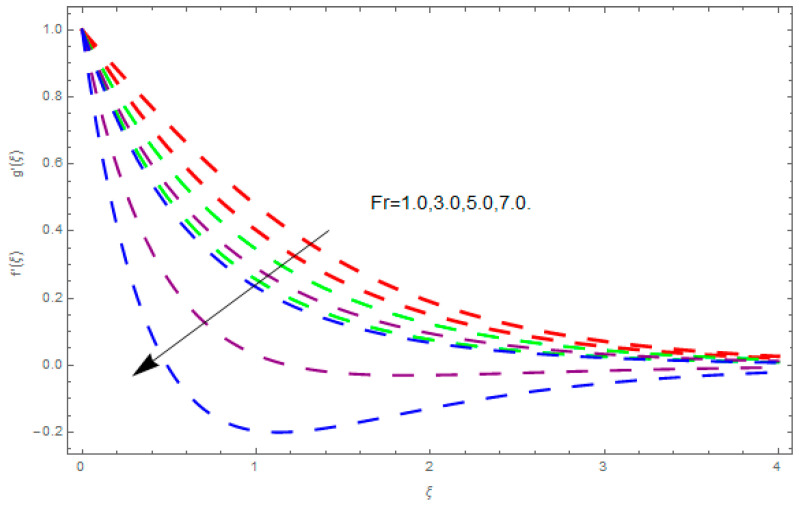
Impression of Fr on $f^{\prime }\left(\xi \right)$ and $g^{\prime }\left(\xi \right)$.

**Figure 7:**
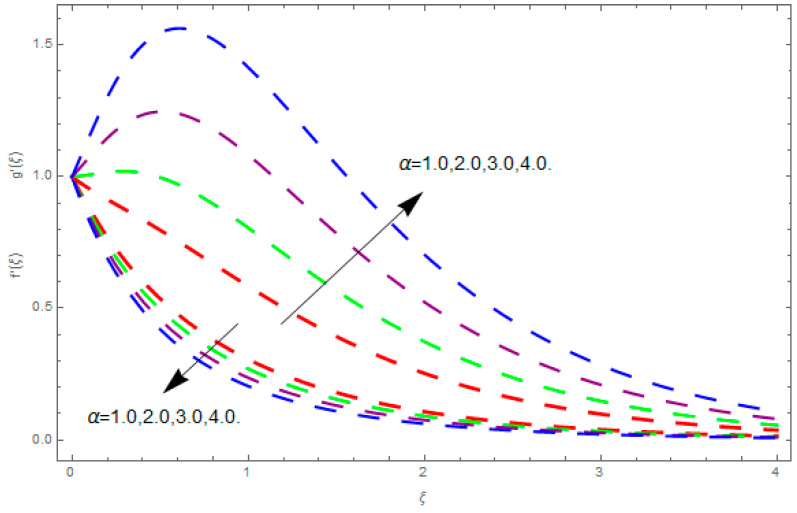
Impression of α on $f^{\prime }\left(\xi \right)$ and $g^{\prime }\left(\xi \right)$.

**Figure 8:**
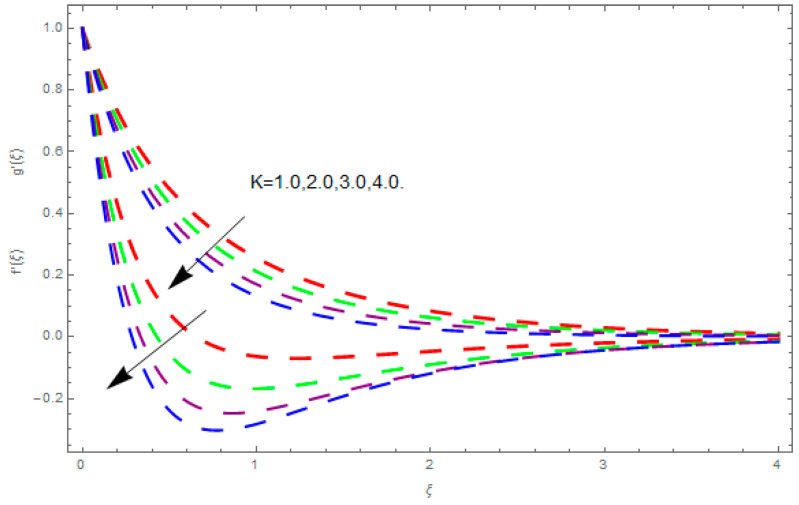
Impression of K on $f^{\prime }\left(\xi \right)$ and $g^{\prime }\left(\xi \right)$.

**Figure 9:**
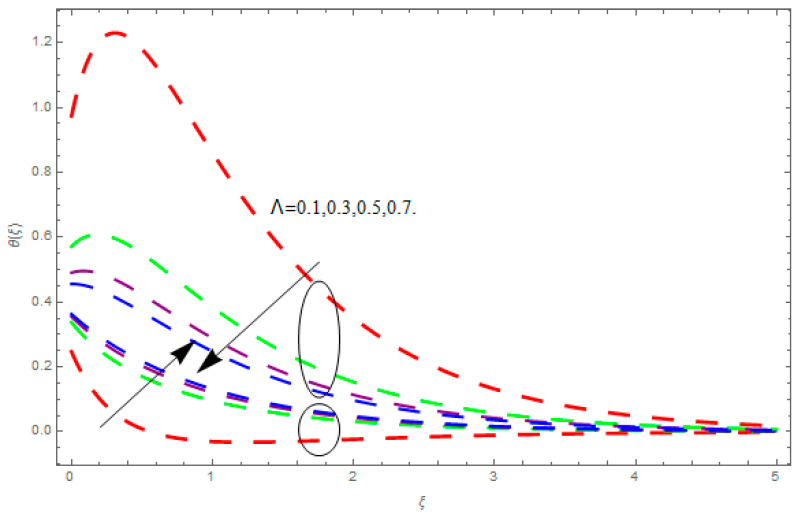
Impression of Λ on θ(ξ).

**Figure 10:**
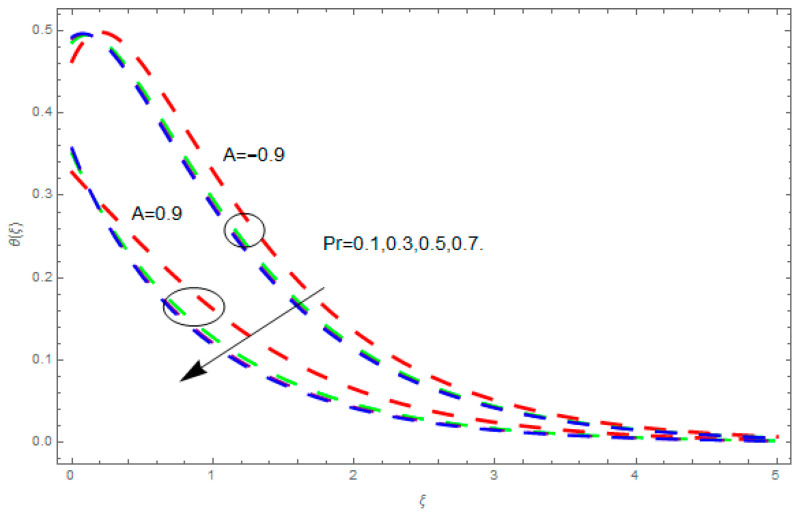
Impression of Pr on θ(ξ).

**Figure 11:**
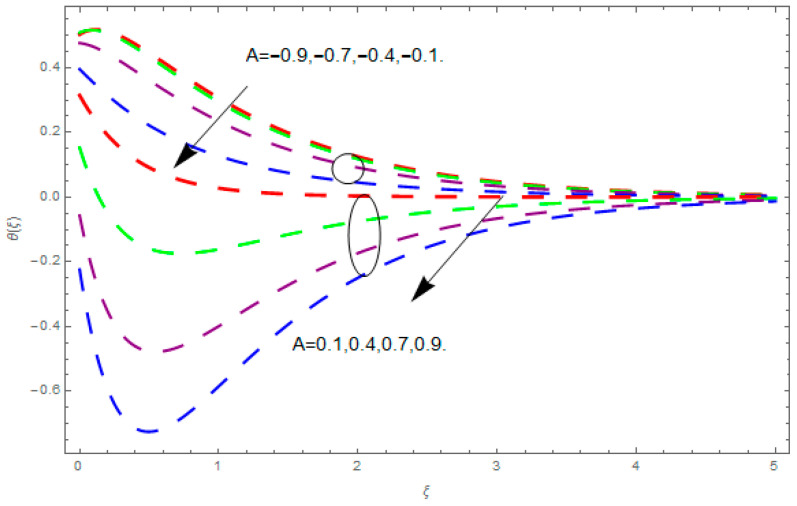
Impression of A on θ(ξ).

**Figure 12:**
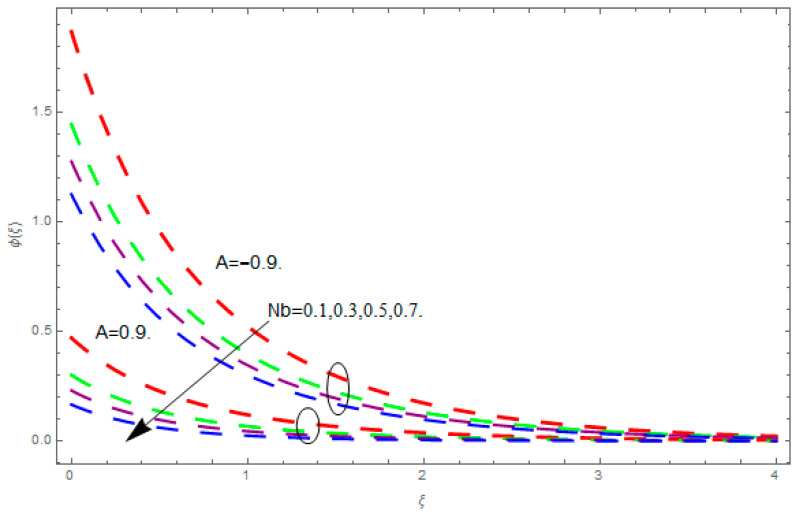
Impression of Nb on ϕ(ξ).

**Figure 13:**
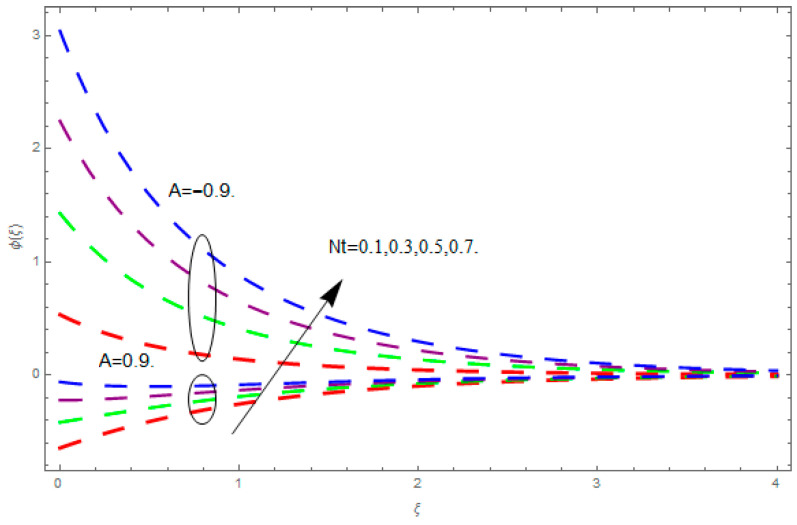
Impression of Nt on ϕ(ξ).

**Figure 14:**
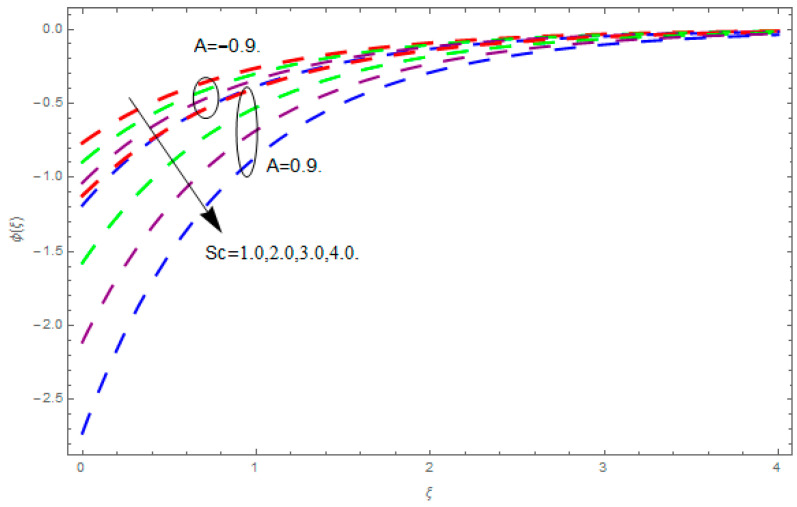
Impression of Sc on ϕ(ξ).

**Figure 15:**
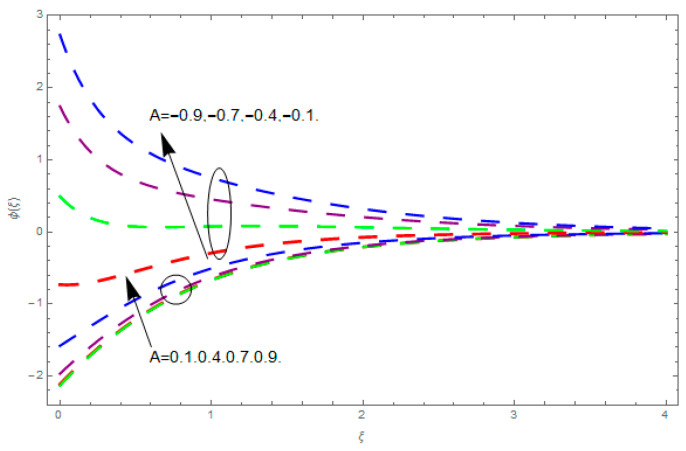
Impression of A on ϕ(ξ).

This section describes the impacts of dimensionless parameters that arose while studying the fluids flow phenomena. These parameters are magnetic field parameter, M, ratio of rates parameter, α, porosity parameter, κ, coefficient of inertia, Fr, couple stress parameter, K, thermal relaxation time, Λ, Brownian motion parameter, Nb, Schmidt number, Sc, and thermophoresis parameter, Nt. The impression of M on $f^{\prime }\left(\xi \right)$ and $g^{\prime }\left(\xi \right)$ is demonstrated in Figure 4. Theory of Lorentz force tells us that escalating M decreases $f^{\prime }\left(\xi \right)$ and $g^{\prime }\left(\xi \right)$. Large values of magnetic field M produce more collisions among molecules, which yield the opposite force to the flow. Therefore, the behavior of fluid flow falls down. The impression of κ on $f^{\prime }\left(\xi \right)$ and $g^{\prime }\left(\xi \right)$ is demonstrated through Figure 5. The porous media plays a significant role in the fluid flow phenomena. The porous media increases the opposing force to fluid flow, which reduces the motion of fluid particles and subsequently the velocity of the fluid reduces. An analogous effect of κ on $g^{\prime }\left(\xi \right)$ is depicted here. The impression of Fr on $f^{\prime }\left(\xi \right)$ and $g^{\prime }\left(\xi \right)$ is revealed in Figure 6. Fr has an inverse relationship with the fluid flow. The increasing Fr reduces the fluid flow motion. This impact is due to the direct relationship of the coefficient of inertia and porous media. As mentioned above, the fluid flow motion reduces in the porous media. The impression of α on $f^{\prime }\left(\xi \right)$ and $g^{\prime }\left(\xi \right)$ is displayed in Figure 7. Large values of α upsurges $f^{\prime }\left(\xi \right)$, but declines $g^{\prime }\left(\xi \right)$. This effect is due to more dominancy in α along the y-direction of the fluid flow, as compared to α in the x-direction. The impression of K on $f^{\prime }\left(\xi \right)$ and $g^{\prime }\left(\xi \right)$ is demonstrated in Figure 8. K is directly proportional to couple stress viscosity parameter n. Larger numerical values of K provides more viscosity of the fluid, which reduces fluid flow and, as a result, reduction in $f^{\prime }\left(\xi \right)$ and $g^{\prime }\left(\xi \right)$ is obtained. The influence of Λ on θ(ξ) is demonstrated through Figure 9. Here, it is noticed that there is an inverse relationship between Λ and θ(ξ). The increasing values of Λ reduces the temperature of the fluid flow. Additionally, Λ versus A is studied. The negative A has an inverse relationship with Λ, while the positive A has a direct relationship with Λ. Thus, the negative A has a dominant impact on the fluid flow. The thermal relaxation time refers to a classical Fourier’s law of conduction. So, it is realized that if temperature is very low than a classical Fourier’s conduction model is obtained. Impact of Pr on θ(ξ) is displayed in Figure 10. Here it is observed that higher numerical values of Pr declines θ(ξ). We deduce from this effect that small numerical values of Pr causes high thermal conductivity, while this effect is quite opposite for large numerical values of Pr. It is important to discuss the impact of A on Pr. The positive A is more effective on Pr as compared to negative A. In addition, negative A plays a dominant role in Pr. The impact of A on θ(ξ) is shown in Figure 11. A and θ(ξ) have an inverse relationship. The escalating A reduces θ(ξ). It is interesting to mention that the positive values of A plays a dominant role in temperature distribution of the fluid flow, as compared to negative values of A. The effect of Nb on ϕ(ξ) is demonstrated through Figure 12. The higher values of Nb boosts the motion of nanoparticles inside the fluid, which results in a reduction of fluid concentration. Thus, large numerical values of Nb reduces ϕ(ξ). It is interesting to mention that the negative values of A plays a dominant role in Brownian motion. The impact of Nt on ϕ(ξ) is portrayed through Figure 13. Large values of Nt increases ϕ(ξ). This is because of the fact that larger values of Nt thrust the nanoparticles of the fluid from the hot region, which results in the increase of ϕ(ξ). It is interesting to mention that the negative A plays a dominant role in Nt. Impact of Sc on ϕ(ξ) is portrayed in Figure 14. Really, the weak diffusivity of mass is noted for higher Sc values. This weak diffusivity of mass has a marvelous effect on fluid concentration, which results in the decrease of ϕ(ξ). Here, in the Schmidt number, the positive A plays a dominant role. The impact of A on ϕ(ξ) is depicted in Figure 15. Here we have an interesting behavior of A. Both the positive and negative A have an increasing behavior in ϕ(ξ). But the negative A is more dominant on concentration as compared to positive A.

## 6. Tables Discussion

Table 1 and Table 2 demonstrate the outcomes of incipient parameters on skin friction coefficients in *x*- and *y*-directions, respectively. Parameters under discussion are ratio of rates parameter, α, couple stress, K, magnetic field, M, porosity, κ, and coefficient of inertia, Fr. We observed that higher ratio of rates, α, couple stress, K, magnetic field, M, and porosity, κ, boost the skin friction coefficient, while higher values of inertia, Fr, falloff the skin friction coefficient. The results were compared with Ramzan et al. [21] and showed a very close agreement.

## 7. Conclusions

The 3D magnetohydrodynamics flow of Darcy–Forchheimer couple stress nanofluid flow over an exponentially stretching sheet is presented. To examine the relaxation characteristics, the proposed model of Cattaneo–Christov is applied. For the first time, the influence of the temperature exponent is explored in the current work.

The concluding remarks are given as:

- The augmented ratio of rates parameter increases the velocity profile in the x-direction.
- The augmented magnetic field, porosity parameter, coefficient of inertia, and couple stress parameter diminishes the velocity field along the x− direction.
- The augmented magnetic field, porosity parameter, coefficient of inertia, ratio of rates parameter, and couple stress parameter reduces the velocity field along the y− axis.
- The enhancement in Prandtl number, time relaxation, and temperature exponent reduces the temperature field.
- The augmented thermophoresis and temperature exponent upsurges the concentration field.
- The augmented Brownian motion and Schmidt number reduces the concentration field.
- The augmented ratio of rates, couple stress, magnetic field, and porosity parameters upsurges the skin friction coefficient.
- The augmented coefficient of inertia diminishes the skin friction coefficient.

## Figures and Tables

**Table 1:** Calculation of skin friction coefficient CfRex1/2 for α,K,M,Fr, and κ.

α	K	M	Fr	κ	Ramzan et al. [21]	Present Study
0.1					1.43588	1.435881
0.2					1.45336	1.453367
0.3					1.51480	1.514801
0.1	0.02				1.48241	1.482410
	0.03				1.58900	1.589002
	0.01	0.1			1.37723	1.377231
		0.2			1.38939	1.389390
		0.3			1.40891	1.408911
		0.1	0.2		-	1.268100
			0.3		-	1.265650
			0.4		-	1.263190
			0.1	0.1	-	1.273000
				0.2	-	1.275451
				0.3	-	1.277892

**Table 2:** Calculation of skin friction coefficient CgRex1/2 for α,K,M,Fr, and κ.

α	K	M	Fr	κ	Ramzan et al. [21]	Current Study
0.1					0.143578	0.143577
0.2					0.299583	0.299582
0.3					0.467421	0.467420
0.4					0.646402	0.646401
0.1	0.02				0.147835	0.147834
	0.03				0.154193	0.154192
	0.04				0.164693	0.164692
	0.01	0.5			0.146938	0.146937
		0.6			0.150971	0.150970
		0.7			0.155608	0.155607
			0.2		-	0.331610
			0.3		-	0.330969
			0.4		-	0.330280
				0.1	-	0.330966
				0.2	-	0.331603
				0.3	-	0.332237

## Nomenclature

A	Temperature exponent
$U_{0},V_{0}$	Constants
${Β}_{0}$	Magnetic field strength $\left({\mathrm{NmA}}^{-1}\right)$
C	Coefficient of concentration
$C_{f}$	Skin friction coefficient
$c_{p}$	Specific heat $\left({\mathrm{Jkg}}^{-1}K^{-1}\right)$
$D_{B}$	Brownian diffusion of nanofluids
$D_{T}$	Thermophoretic diffusion of nanofluids
E	Electric field $\left({\mathrm{NC}}^{-1}\right)$
f,g	Dimensional velocity profiles
K	Couple stress parameter
L	Reference length (m)
M	Hartmann number
Nb	Brownian motion
Nt	Thermophoretic parameter
$Νu_{x}$	Nusselt number
Pr	Prandtl number
$q_{r}$	Heat flux $\left({\mathrm{Wm}}^{-2}\right)$
Rex	Local Reynolds number
Sc	Schmidt number
$Sh_{x}$	Sherwood number
T	Fluid temperature (K)
u,v,w	Velocity components $\left({\mathrm{ms}}^{-1}\right)$
x,y,z	Coordinates
$y_{i}(i=1-10)$	Constants
**Greek Letters**	
α	Ratio of rates parameter
Λ	Thermal relaxation time
γ	Biot number
θ	Dimensional heat profile
Φ	Dimensional concentration profile
ξ	Similarity variable
ν	Kinematic viscosity $\left(m^{2}s^{-1}\right)$
κ	Porosity parameter
ρ	Fluid density $\left({\mathrm{Kgm}}^{-3}\right)$
$\sigma_{nf}$	Electrical conductivity $\left({\mathrm{Sm}}^{-1}\right)$
